# Circumscribed Morphea Successfully Treated With Excimer Laser: Analysis of Histopathological Changes

**DOI:** 10.1111/1346-8138.70225

**Published:** 2026-03-18

**Authors:** Takuya Takahashi, Takehiro Takahashi, Yuichiro Segawa, Kenta Oka, Toshiya Takahashi, Yoshihide Asano

**Affiliations:** ^1^ Department of Dermatology Tohoku University Graduate School of Medicine Sendai Japan

**Keywords:** circumscribed morphea, excimer laser, localized scleroderma, phototherapy

## Abstract

Localized scleroderma (LSc) is an autoimmune condition characterized by localized cutaneous sclerosis, sometimes extending into deeper tissues. Phototherapy, including excimer laser therapy (ELT), is considered an effective and minimally invasive treatment option for patients without extracutaneous involvement. However, little is known about the histopathological and molecular alterations that occur during treatment. Here, we report a case of circumscribed morphea successfully treated with ELT, accompanied by detailed longitudinal histological analysis. A 78‐year‐old woman presented with gradually progressive indurated plaques on both sides of the abdomen. Histopathological examination of the lesional skin revealed dense collagen deposition and perivascular infiltration of lymphocytes, monocytes, and mast cells, confirming the diagnosis of circumscribed morphea. Topical glucocorticoid therapy yielded insufficient improvement, prompting the addition of 308‐nm ELT. The patient underwent 12 ELT sessions, administered every two to four weeks with incremental dosing from 100 mJ/cm^2^ to 240 mJ/cm^2^, reaching a cumulative dose of 2100 mJ/cm^2^. This regimen resulted in marked clinical improvement within one year. Post‐treatment biopsies demonstrated near‐complete resolution of dermal sclerosis, with substantial reductions in inflammatory cell infiltration. Toluidine blue staining and immunohistochemistry further revealed dynamic cellular changes: mast cells and CD3^+^ T cells were significantly decreased; CD34 expression, absent in lesional dermal mesenchymal cells before treatment, was restored; and α‐smooth muscle actin‐positive myofibroblasts, abundant at baseline, were markedly reduced following ELT. These findings indicate that ELT not only ameliorates clinical sclerosis but also reverses immune and mesenchymal cell alterations associated with fibrosis. This case highlights the therapeutic potential of ELT in circumscribed morphea and suggests a plausible mechanism by which ELT modulates immune–mesenchymal interactions to attenuate fibrosis in LSc.

## 1 Introduction

Localized scleroderma (LSc) is an autoimmune disorder marked by cutaneous sclerosis confined to specific areas and may involve deeper structures such as subcutaneous tissue, bone, or the central nervous system [1]. LSc is classified into five subtypes: circumscribed morphea, linear scleroderma, generalized morphea, pansclerotic morphea, and mixed morphea [2]. The clinical course typically progresses through three phases: inflammatory, sclerotic, and atrophic [1]. Regarding LSc treatment, topical and systemic therapies, as well as phototherapy, have been proposed [3]. Systemic therapies are preferred for patients with extracutaneous involvement, whereas topical treatments and phototherapy are suitable for those without, given their low risk of systemic adverse effects [3].

The excimer laser, which emits monochromatic light at 308 nm, is used to treat various dermatological conditions, including vitiligo, psoriasis, and lichen planus [4]. Its mechanism of action is thought to induce DNA damage in keratinocytes and T cells, reducing local inflammation and decreasing keratinocyte activity [5]. Previous studies have demonstrated the efficacy of excimer laser therapy (ELT) in treating LSc [4, 5]. However, data on the molecular and histopathological dynamics throughout this treatment remain limited. Here, we present a case successfully treated with ELT, in which we analyzed longitudinal changes of molecular markers associated with skin sclerosis of LSc.

## 2 Case Report

**FIGURE 1:**
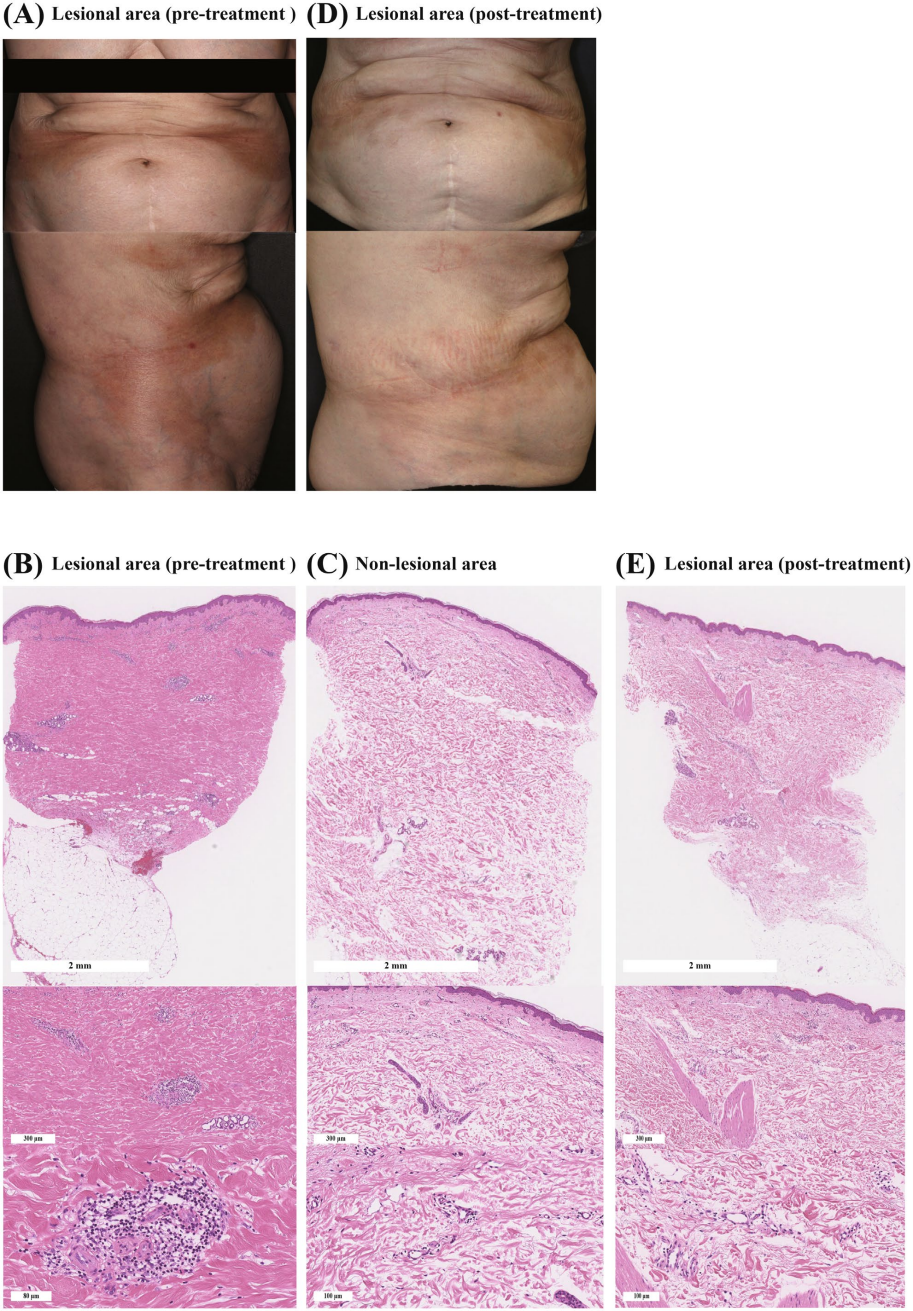
Clinical and histopathological findings. (A) Clinical findings at the initial visit showing indurated, patchy plaques with erythema, a shiny surface, and pigmentation on both sides of the abdomen. (B, C) Skin biopsies from the lesional abdominal area (B) and the adjacent non‐lesional area (C). The lesional area exhibited dense, diffuse proliferation of coarse collagen fibers throughout the dermis and increased infiltration of inflammatory cells in perivascular and interstitial areas compared with the non‐lesional area. (D) Clinical appearance one year after initiation of excimer laser therapy (ELT), showing improved cutaneous sclerosis with hyperpigmentation. (E) Post‐ELT skin biopsy showing near‐complete resolution of thickened collagen fibers and reduced numbers of infiltrating immune cells.

A 78‐year‐old woman presented with cutaneous induration on both sides of the abdomen. She had no relevant medical history. The induration had developed gradually over the past year. She was treated with topical glucocorticoid ointment; however, the response was insufficient. At presentation, indurated, patchy plaques with erythema, a shiny surface, and pigmentation were observed bilaterally on the abdomen (Figure 1A). Blood tests showed an antinuclear antibody titer of 1:40 by indirect immunofluorescence, white blood cell count (5600/μL: neutrophil 60.9%, lymphocyte 29.9%, eosinophil 0.16%), and creatinine 0.73 mg/dL. Anti‐single stranded DNA (ssDNA) antibody and systemic sclerosis‐associated autoantibodies, including anti‐topoisomerase I antibody, anti‐centromere antibody, anti‐RNA polymerase III antibody, and anti‐U1 ribonucleoprotein antibody, were all negative. Skin biopsies from lesional (Figure 1B) and non‐lesional areas (Figure 1C) revealed dense collagen deposition and perivascular infiltration of lymphocytes, monocytes, and mast cells in the lesional dermis (Figure 1B). Given these manifestations, we diagnosed her with circumscribed morphea. Topical glucocorticoid ointment was continued, but sclerosis did not sufficiently improve. Therefore, ELT using XTRAC (STRATA Skin Sciences Inc.) was added to the topical treatment, starting at 100 mJ/cm^2^ and gradually increased every two to four weeks up to 240 mJ/cm^2^, for a total of 12 sessions with a cumulative dose of 2100 mJ/cm^2^. Following the initiation of combination therapy, cutaneous sclerosis resolved a year after ELT treatment introduction (Figure 1D). At the 12th session (1 year after ELT initiation), a biopsy was performed to evaluate remaining disease activity. Histological analysis revealed that the previously observed thickened collagen fibers throughout the dermis had nearly disappeared (Figure 1E). In addition, the number of perivascular inflammatory cells was markedly reduced (Figure 1E). These histological changes were consistent with the clinical improvement.

**FIGURE 2:**
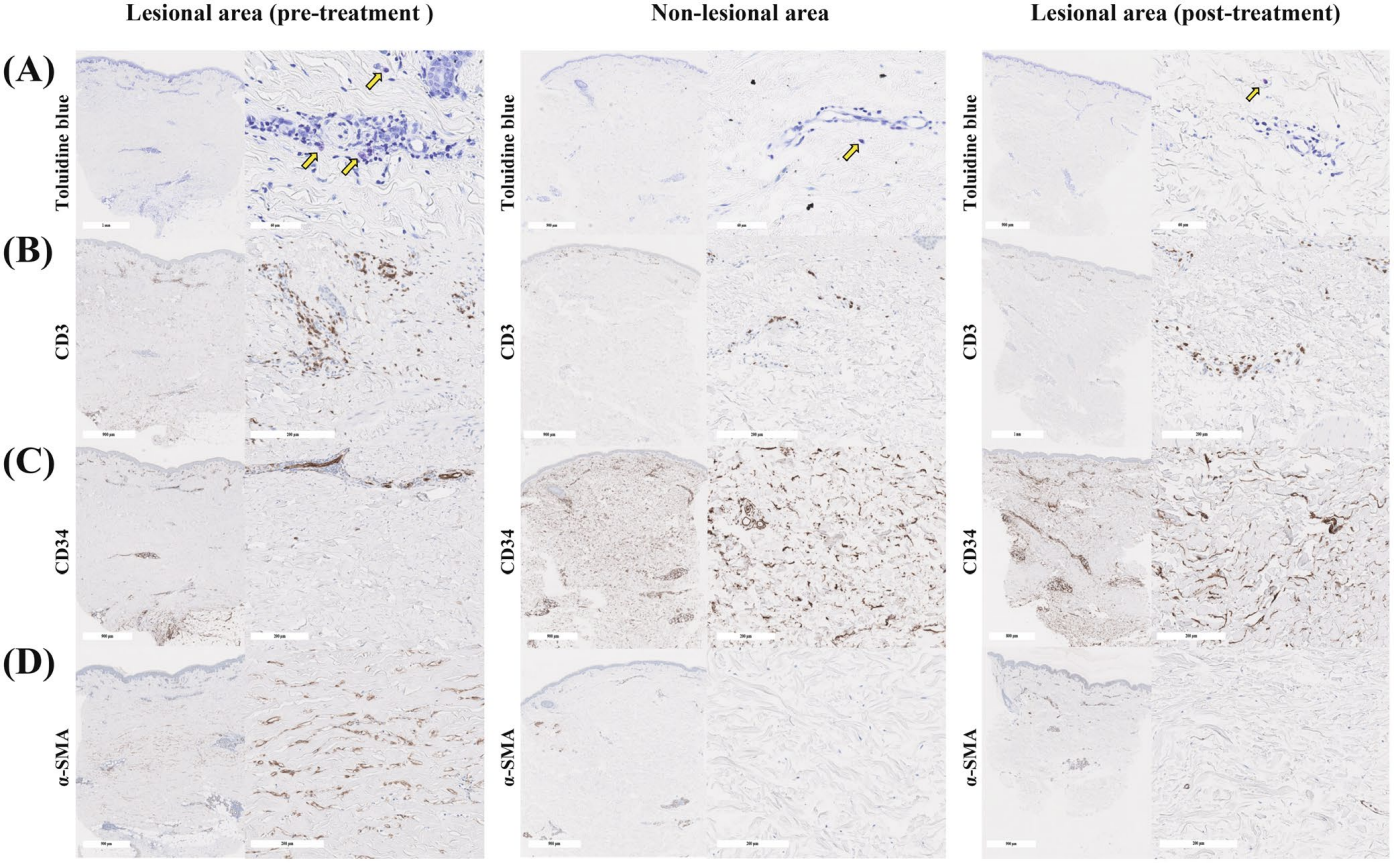
Histological and immunohistochemical (IHC) analysis of abdominal skin. Skin biopsies were obtained from the lesional area before ELT, the non‐lesional area, and the lesional area after ELT. (A) Toluidine blue staining revealed increased numbers of metachromatically stained cells (yellow arrows) in the pre‐treatment lesional area (left panels). IHC staining showed increased CD3^+^ cells (B), absence of CD34^+^ mesenchymal cells (C), and increased numbers of α‐SMA‐positive cells (D) in the lesional dermis (right panels) compared with the non‐lesional area (middle panels). In contrast, all of these abnormalities were reversed in the post‐treatment lesional area (right panels of A–D).

To further characterize histological features, we conducted Toluidine blue staining and immunohistochemical staining for CD3, CD34, and α‐smooth muscle actin (α‐SMA). Toluidine blue staining revealed increased mast cells in perivascular and mesenchymal regions of the lesional dermis compared with the non‐lesional dermis (Figure 2A). The number of CD3^+^ cells was higher in the lesional dermis than in the non‐lesional dermis (Figure 2B). Both of these infiltrating immune cells were markedly reduced after ELT. CD34 expression was absent in lesional dermal mesenchymal cells but present in those of the non‐lesional mesenchymal cells (Figure 2C). In contrast, α‐SMA was highly expressed in lesional fibroblasts but undetectable in non‐lesional dermis (Figure 2D). Following ELT, these alterations reversed to the levels comparable to the non‐lesional dermis, as evidenced by the recovery of CD34 staining and clear reduction of α‐SMA‐positive fibroblasts (Figure 2C,D).

## 3 Discussion

Although UVA1 treatment has historically been preferred over UVB radiation for LSc because of its greater dermal penetration, our case and previous reports demonstrate that ELT improves the dermal sclerosis despite its limited penetration into the dermis [3, 5]. Recent single‐cell RNA sequencing studies have revealed complex interactions among keratinocytes, fibroblasts, and immune cells in the pathogenesis of LSc [6]. These findings suggest that targeting keratinocyte–immune cell crosstalk may be critical in modulating disease progression [7]. UVB therapy is suggested to suppress keratinocyte activity, thereby reducing the secretion of pro‐inflammatory factors such as IL‐1α and limiting interactions with T cells, B cells, and macrophages, thereby ultimately attenuating fibroblast activation and excessive collagen production in the dermis [5, 6, 7].

Besides keratinocyte‐mediated pathways, immune and mesenchymal cell populations also contribute to dermal fibrosis in LSc. Prior reports show that mast cells contribute to dermal fibrosis in LSc, with increased numbers in lesional areas correlating with collagen accumulation [8]. The development of fibrosis involves mast cell‐mediated differentiation of fibroblasts into myofibroblasts, which produce excessive ECM [8]. T cell numbers increase during both the inflammatory and fibrotic phases, and these infiltrating T cells promote LSc progression [1]. Furthermore, the extent of α‐SMA expression negatively correlates with mesenchymal CD34 positivity, both serving as markers of dermal fibrosis [9]. Recently, a subset of CD34‐positive dermal mesenchymal cells, termed telocytes, has been identified as key regulators of tissue homeostasis, whose loss results in tissue fibrosis [10]. The observed negative correlation between α‐SMA and CD34 indicates depletion of telocytes and/or their transition into myofibroblasts [9, 10] Collectively, mast cell and T cell infiltration, α‐SMA upregulation, and the loss of CD34‐positive mesenchymal cells, possibly including telocytes, drive dermal fibrosis and reflect LSc severity.

In our case, the pre‐treatment lesional area displayed increased mast cell and T cell counts, CD34 negativity, and enhanced α‐SMA expression, consistent with progressive LSc. Following ELT, all parameters returned to non‐lesional levels, demonstrating histological improvement and supporting its therapeutic efficacy for dermal sclerosis.

From a practical clinical perspective, UVB therapy offers several advantages over UVA1 therapy. The treatment time required for ELT is substantially shorter than that for UVA1 therapy, allowing large areas to be treated within a substantially shorter time [11]. Furthermore, unlike systemic treatments such as corticosteroids and methotrexate, ELT is associated with a considerably lower risk of systemic adverse events, including endocrine and musculoskeletal complications [12, 13]. While severe cases with extracutaneous involvement would more likely necessitate systemic treatments, ELT may represent a suitable therapeutic option for patients with LSc without extracutaneous involvement, including those with circumscribed morphea. In our case, the sclerotic lesions were too extensive to be treated efficiently with UVA1 therapy within a reasonable treatment time at the outpatient clinic, whereas ELT sessions required only several minutes, thereby minimizing treatment burden. In addition, as the patient did not demonstrate extracutaneous involvement, systemic therapy was not considered necessary. Therefore, ELT combined with topical corticosteroids was considered to be a good therapeutic option for this patient.

Considering the therapeutic course in our case retrospectively, topical glucocorticoid therapy alone was insufficient to improve her indurative lesions; however, clinical manifestations improved after the addition of ELT. A network meta‐analysis of UVB‐based therapy for vitiligo vulgaris demonstrated that combination therapy with topical corticosteroids and phototherapy achieved superior clinical outcomes compared with phototherapy alone [14]. Although similar evidence in patients with LSc has been lacking, the combination of topical corticosteroids with ELT may provide an additive therapeutic effect in selected patients with morphea.

In summary, we report a case of circumscribed morphea successfully treated with ELT, which was in parallel with the histopathological changes at the molecular level. Further research is warranted to elucidate its underlying mechanisms and confirm its efficacy across different LSc subtypes.

## Disclosure

Declaration of generative AI and AI‐assisted technologies in the writing process: During the preparation of this manuscript, the author utilized ChatGPT (*GPT‐5* (OpenAI, 2025)) to improve the clarity and language of the text. All content was subsequently reviewed and edited by the authors. The authors take full responsibility for the final version of the manuscript.

## Funding

The authors have nothing to report.

## Ethics Statement

Written informed consent was obtained from the patient for publication of this case report and the accompanying clinical images.

## Conflicts of Interest

The authors declare no conflicts of interest.

## Data Availability

The data that support the findings of this study are available from the corresponding author upon reasonable request.

## References

[1] C. Papara , D. A. De Luca , K. Bieber , A. Vorobyev , and R. J. Ludwig , “Morphea: The 2023 Update,” Frontiers in Medicine (Lausanne) 10 (2023): 1108623, 10.3389/fmed.2023.1108623. PMC9969991 36860340

[2] R. M. Laxer and F. Zulian , “Localized Scleroderma,” Current Opinion in Rheumatology 18, no. 6 (2006): 606–613, 10.1097/01.bor.0000245727.40630.c3. 17053506

[3] A. Kreuter , “Localized Scleroderma,” Dermatologic Therapy 25, no. 2 (2012): 135–147, 10.1111/j.1529-8019.2012.01479.x. 22741933

[4] S. Mehraban and A. Feily , “308nm Excimer Laser in Dermatology,” Journal of Lasers in Medical Sciences 5, no. 1 (2014): 8–12. 25606333 PMC4290518

[5] P. Szczepanik‐Kułak , M. Michalska‐Jakubus , and D. Krasowska , “Laser Therapy for the Treatment of Morphea: A Systematic Review of Literature,” Journal of Clinical Medicine 10, no. 15 (2021): 3409, 10.3390/jcm10153409. 34362192 PMC8347526

[6] A. B. Rosen , A. Sanyal , T. Hutchins , et al., “Unique and Shared Transcriptomic Signatures Underlying Localized Scleroderma Pathogenesis Identified Using Interpretable Machine Learning,” JCI Insight 10, no. 7 (2025): e185758, 10.1172/jci.insight.185758. 40197368 PMC11981619

[7] J. Yoshimura , Y. Asano , T. Takahashi , et al., “A Case of Scleredema Adultorum Successfully Treated With Narrow‐Band Ultraviolet B Phototherapy,” Modern Rheumatology 26, no. 2 (2016): 302–306, 10.3109/14397595.2013.875640. 24499427

[8] K. Takeda , A. Hatamochi , and H. Ueki , “Increased Number of Mast Cells Accompany Enhanced Collagen Synthesis in Linear Localized Scleroderma,” Archives of Dermatological Research 281, no. 4 (1989): 288–290, 10.1007/bf00431065. 2774661

[9] J. S. Lee , H. S. Park , H. S. Yoon , J. H. Chung , and S. Cho , “CD34 Stromal Expression Is Inversely Proportional to Smooth Muscle Actin Expression and Extent of Morphea,” Journal of the European Academy of Dermatology and Venereology 32, no. 12 (2018): 2208–2216, 10.1111/jdv.15120. 29888507

[10] I. Rosa , E. Romano , B. S. Fioretto , and M. Manetti , “Pathophysiologic Implications and Therapeutic Potentials of Telocytes in Multiorgan Fibrosis,” Current Opinion in Rheumatology 38, no. 1 (2025): 26–37, 10.1097/bor.0000000000001116. 40747598 PMC12672042

[11] I. M. Majoie , J. M. Oldhoff , H. van Weelden , et al., “Narrowband Ultraviolet B and Medium‐Dose Ultraviolet A1 Are Equally Effective in the Treatment of Moderate to Severe Atopic Dermatitis,” Journal of the American Academy of Dermatology 60, no. 1 (2009): 77–84, 10.1016/j.jaad.2008.08.048. 19103360

[12] E. B. Kroft , M. C. Creemers , F. H. van den Hoogen , J. B. Boezeman , and E. M. de Jong , “Effectiveness, Side‐Effects and Period of Remission After Treatment With Methotrexate in Localized Scleroderma and Related Sclerotic Skin Diseases: An Inception Cohort Study,” British Journal of Dermatology 160, no. 5 (2009): 1075–1082, 10.1111/j.1365-2133.2008.09017.x. 19210503

[13] R. S. Hardy , K. Raza , and M. S. Cooper , “Therapeutic Glucocorticoids: Mechanisms of Actions in Rheumatic Diseases,” Nature Reviews Rheumatology 16, no. 3 (2020): 133–144, 10.1038/s41584-020-0371-y. 32034322

[14] Y. Wen , I. A. Issa , L. Lei , et al., “Impacts of Topical Treatments and Phototherapy on Stratum Corneum Hydration, Sebum and Elasticity in Vitiligo Skin,” Dermatol. Ther. (Heidelb.) 15, no. 5 (2025): 1163–1171, 10.1007/s13555-025-01398-y. 40172744 PMC12033151

